# Morphine concentrations in fatalities after palliative treatment of acute burn injury

**DOI:** 10.1007/s00414-024-03164-9

**Published:** 2024-01-17

**Authors:** Julian Bickel, Nadine Aboutara, Hilke Jungen, Anne Szewczyk, Alexander Müller, Benjamin Ondruschka, Stefanie Iwersen-Bergmann

**Affiliations:** 1 Department of Legal Medicine, University Medical Centre Hamburg-Eppendorf, Hamburg, Germany; 2 Research Centre Borstel Leibniz Lung Centre, Borstel, Germany

**Keywords:** Morphine concentrations, Palliative treatment, Acute burn injury, Postmortem analysis, Forensic toxicology, Pharmacokinetic modelling

## Abstract

The evaluation of a morphine concentration in postmortem blood is routine for a forensic toxicologist. We here report three fatal cases where we found high morphine concentrations with 7.96, 4.30, and 5.82 mg/l in femoral blood that have to be estimated as unusually high. All these individuals died due to severe burn injuries and obtained morphine in the context of their palliative care in the last hours of their lives. According to the autopsy results, the cause of death in case 1 was burn disease with burns of about 90% of the body surface area (BSA), case 2 burn trauma, and case 3 burn shock. Besides morphine, propofol, fentanyl, sufentanil, midazolam, diazepam, lorazepam, cefazolin, and rocuronium were detected in femoral blood. The findings fitted well with the detailed clinical documentation. Further evidence of therapeutic concentrations of quetiapine, duloxetine, and melperone could be matched to preexisting medication of the individuals. Physiologically based pharmacokinetic modelling (PBPK) was applied, developed for the intravenous administration of morphine, to find an explanation for the high morphine concentrations in femoral blood. Quantification of morphine in body fluids and tissue was performed to calculate morphine tissue concentration ratios to the morphine concentration in femoral blood. The presented cases show that pharmacokinetic simulations can reflect decreased renal clearance and decreased hepatic metabolism in general. However, this prediction is not sufficient to explain the high morphine concentrations in femoral blood measured here. It can be assumed that burn shock in particular leads to altered pharmacokinetics, namely decreased distribution of morphine.

## Introduction

The evaluation of drug concentrations in postmortem specimens is an important task in forensic toxicology. In particular, the opioid group, commonly used for pain management, exhibits toxic effects in case of overdose typically manifested as respiratory depression. Although oxycodone, hydromorphone, hydrocodone, tapentadol, and fentanyl are increasingly being used, morphine remains an important analgesic [1, 2]. Morphine is typically used to treat the pain of seriously ill patients. If they die in the course of treatment, the question arises as to whether the prescribed dosage was too high or whether more morphine was administered than medically prescribed. The evaluation of a morphine concentration in postmortem blood is therefore a question that a forensic toxicologist is occasionally confronted with.

In comparison to serum concentrations in living persons, morphine concentrations in the blood of a deceased are prone to alterations already during the pre-final phase due to altered pharmacokinetics. It is therefore important to fully understand the pathology and circumstances of death in order to evaluate morphine concentrations measured postmortem. However, potential postmortem changes must be considered as well. Data compilations of blood concentrations in postmortem cases [3] are really helpful for the assessment of morphine blood concentrations, but especially in particular case constellations, case reports with similar constellations can be of additional help.

We report three fatalities in severely burn injured individuals who were given morphine in palliative care in the last hours of their lives. Although reviews occasionally report that deaths after burn injury show higher morphine concentrations [4], specific reports of this phenomenon and its potential reason are scarce.

### Case Report

The following case reports are based on clinical documentation and full forensic autopsy protocols. Details of midazolam and morphine dosing in each case are summarized in Tables 1, 2, and 3.

**Table 1:** Morphine application of case 1; the drug dose of morphine sulphate was converted to morphine base

Case 1				
Duration	Drug	Application	Infusion rate	Dose
1:59 h	Morphine	Infusion	37.6 mg/h	74.0 mg

**Table 2:** Morphine application of case 2; the drug dose of morphine sulphate was converted to morphine base

Case 2				
Duration	Drug	Application	Infusion rate	Dose
4:48 h	Morphine	Infusion	37.6 mg/h	179.8 mg
0:21 h	Morphine	Infusion	48.9 mg/h	22.9 mg

**Table 3:** Morphine application of case 3; the drug dosage of morphine sulphate was converted to morphine base

Case 3				
Duration	Drug	Application	Infusion rate	Dose
2:37 h	Morphine	Infusion	37.6 mg/h	98.9 mg

### Case 1

A 78-year-old woman was rescued from a burning house that had exploded. She sustained severe burns and was diagnosed in the hospital with third-degree burns covering 85% of her body surface area (BSA) and second-degree b burns covering 5% BSA, but without inhalation trauma. A debridement surgery was performed, but given the low chance of survival, palliative therapy was initiated 1 h and 40 min before she died. Morphine was administered via a perfusor inserted in the left femoral vein for around 2 h. The infusion was stopped 18 min after her death. During the therapy, 1.5 L of an isotonic electrolyte solution was administered also. Estimated glomerular filtration rate (eGFR) was reported at the hospital 2 h and 10 min before her death as 28 ml/min/1.73 m^2^.

#### Autopsy Findings

Autopsy was performed 3 days after death. It revealed signs of direct fire exposure with extensive burns of second degree to fourth degree nearly of the entire surface of the body. Head hair, eyelashes, and eyebrows were scorched; carbon black deposits in the upper respiratory tract were detected too as sign of vitality. Further findings were cerebral swelling, congested lungs, and pathological cardiac enlargement due to arterial hypertension. The autopsy report concluded that the cause of death was burn disease with burns of about 90% BSA.

### Case 2

After being rescued from his burning apartment, a 44-year-old man was diagnosed with third-degree burns to 75% of his BSA and inhalation trauma, for which he underwent an escharotomy and debridement surgery in the hospital. Palliative therapy with morphine was administered via a perfusor 5 h and 7 min before he died. The infusion rate had to be increased after 4 h and 48 min due to his tachypnoea and tachycardia, and the therapy was stopped 2 min after his death. eGFR was reported at the hospital 6 h and 6 min before his death as 30 ml/min/1.73 m^2^.

#### Autopsy findings

Autopsy was performed 7 days after death. It revealed signs of direct heat exposure with extensive burns of second degree to fourth degree especially to the front of the body, blackish fluid in the respiratory tract, carbon black suspicious deposits on the epiglottis, and on the mucous membrane of the larynx and trachea, as well as in the esophagus and in the contents of the stomach as inhalation signs. Further findings were cerebral swelling, congested lungs, and pathologic cardiac enlargement. The autopsy report concluded that the cause of death was burn trauma.

### Case 3

An 83-year-old woman was rescued from an explosion site and received midazolam i.v. during transport to the hospital. There she was diagnosed with a 72% BSA burn, consisting of 15% second-degree a, 34% second-degree a/b, and 23% third-degree burns, without inhalation trauma. She underwent debridement surgery, and palliative therapy was started with morphine via perfusor 2 h and 42 min before her death. The infusion was stopped 5 min before she died. During the therapy, a total of 4.2 L of isotonic electrolyte solution were administered. eGFR was reported at the hospital 5 h and 51 min before her death as 42 ml/min/1.73 m^2^.

#### Autopsy findings

Autopsy was performed 10 days after death. It revealed signs of direct heat exposure with extensive burns of second degree and third degree of the BSA, particularly on the front of the body. Several sharp skin lesions, a few millimetres to several centimetres long, were found on the neck, on the chest, and on the upper and lower extremities. They were evaluated as injuries caused by explosive projectiles, such as flying glass fragments in the explosion room. Further findings were thickened airways covered with carbon black plaques up to the periphery, hyperinflation of lung tissue, scarce lividity, and pale organ colour in the sense of burn anaemia.

In addition, there were shock signs of the kidneys, relative richness of blood of central organs in general anaemia, and relative brain swelling in generalized brain atrophy. The autopsy report concluded that the cause of death was a burn shock.

## Material and Methods

### Samples

The corpses were consequently stored at 4 °C from the admission to our morgue until autopsy. Body fluid and organ samples were collected during the autopsies; they were kept at − 20 °C until analysis.

### Chemical and Reagents

All solvents and reagents for sample preparation were of analytical grade (p.a.) and were obtained from Merck Schuchard (Hohenbrunn, Germany). Solvents and chemicals for LC–MS/MS analysis (water, acetonitrile, formic acid) were of specified LC/MS grade (Chromasolv®) and purchased from Fluka (Munich, Germany). Stock solutions (1.0 mg/ml in methanol) of morphine and morphine D3 were obtained from Lipomed (Weil am Rhein, Germany). Quality control samples for morphine in blood were purchased from ACQ Science GmbH (Rottenburg-Hailfingen, Germany).

### Methods

In all three cases, a full-scale toxicological investigation of all available specimens was performed using immunological methods as well as GC–MS and LC–MS/MS methods.

### Immunoassay

Blood samples were analysed for the presence of amphetamine, substances of the ecstasy group, opiates, cocaine, methadone, ethanol, benzodiazepines, tricyclic antidepressants, oxycodone, and cannabinoids using standard immunoassay screening tests (CEDIA DAU, Thermo Fischer Scientific, Middletown, USA) on an automatic analyser (AU 480) (Beckmann Coulter GmbH, Krefeld, Germany).

### Analysis of Morphine

Morphine was determined as follows. A 0.05 ml aliquot of the blood sample was spiked with 5 µl of the deuterated internal standard solution (0.1 ng/µl), followed by protein precipitation with 250 µl ice cold acetonitrile. After being shaken and centrifugated, 100 µl of the supernatant was diluted with 500 µl water. The resulting solution was analysed via LC–MS/MS (Waters Acquity® UPLC with a C18 Waters Acquity BEH, 1.7 µm, 2.1 × 50 mm column at 40 °C). The injection volume of the sample was 10 µl, the flow rate at 0.5 ml/min, and the binary gradient was as follows: 0–3.0 min: 100% A, 3.0–4.0 min 10% A 90% B, 4.1–5.5 min 100% A. Mobile phase A consisted of 90% 10 mM ammonium bicarbonate in water (pH 9) and 10% methanol; mobile phase B was 100% methanol. A Waters Xevo® TQ-XS triple quadrupole mass analyzer operating in multiple reaction monitoring mode was used for detection (Waters, Milford, USA). This method has been validated according to the current guidelines of the Society for Toxicological and Forensic Chemistry [5]. It has been applied in routine analysis for several years. Regular external quality control is performed by periodical proficiency testing.

In all cases, an additional morphine analysis was performed after enzymatic cleavage (EC) of morphine glucuronides. For this purpose, a 0.2 ml blood sample was incubated with 10 µl beta-glucuronidase (Roche Diagnostics GmbH, Mannheim, Germany) for 2 h at 47 °C. After vortexing and centrifugation, 0.05 ml of the sample was analysed as described above. To evaluate the cleavage capacity of our enzymatic approach, regular testing is conducted using an external control provided by UTAK Laboratories, California, USA. These controls contain morphine-3-glucuronide, and our enzymatic cleavage consistently produces reliable results for free morphine.

Additionally, morphine was analysed in heart blood, liver, kidney, brain tissue, and cerebrospinal fluid in all cases, too. Organ samples were weighed into a grinding tube (IKA GmbH, Staufen im Breisgau, Germany) and homogenized with water (1:1 w/w). After centrifugation, 0.05 ml of the supernatant was prepared as described for blood. Resulting concentrations were doubled to account for the dilution.

### Pharmacokinetic Models

In the three fatalities, high levels of morphine were determined in the femoral blood. Therefore, the pharmacokinetics of morphine in severe burns should be assessed using a pharmacokinetic model. For this purpose, the physiologically based pharmacokinetic modelling (PBPK) was chosen, employing the PBPK software PK-Sim and MoBi (V11, Bayer Technology Services, Leverkusen, Germany), along with predefined anatomical and physiological parameters [6–8].

Schaefer et al. [9] developed the PBPK for the intravenous administration of morphine. The authors investigated whether postmortem analysis of blood and urine morphine concentrations matched clinically documented morphine administration. They successfully validated this model, demonstrating its predictive capability for morphine concentration. This model employed CYP3A4 and UGT2B7 enzymes for morphine metabolism, utilizing reference concentrations of 1 µmol/l (Table 4).

**Table 4:** Morphine parameters used in the simulations: *logP*, octanol–water partition coefficient; *M*, molar mass; *Fub*, unbound fraction; *pKa*, acid dissociation constant; *Cl*, clearance; *GFR*, glomerular filtration rate

Morphine	logP	M [g/mol]	F_ub_ [%]	pK_a_	Solubility [mg/l]	Cl (UGT2B7) [l/min]	Cl (CYP3A4) [l/min]	GFR fraction
	0.9	285.34	65	8.21	149.0	1.47	0.64	1

Three simulations were conducted per case. In the first simulation, renal elimination was set to the eGFR reported by the hospital prior to death. In the next simulation, the metabolism of morphine was disabled. Disabling the metabolism and the renal clearance of morphine was the third simulation.

Table 4 summarizes the pharmacokinetic parameters of morphine as reported [9], while anthropometric and retention parameters of each individual are given in Table 5.

**Table 5:** Individual parameters of each person. *eGFR* estimated glomerular filtration rate

Case	Sex	Age	Ethnicity	Weight [kg]	Height [cm]	eGFR [*ml/min/1.73 m*^*2*^*]*
1	Female	78	European	78.8	148	28
2	Male	44	European	88.3	185	30
3	Female	83	European	50.0	169	42

## Results

Opiates and benzodiazepines were detected in all subjects with immunoassay screening of femoral blood. Co-Hb levels were below 10% in all cases, which was not surprising looking at the survival times. Quantitative results of morphine in blood are shown in Table 6. Ratio of morphine concentrations in tissue material to femoral blood morphine concentrations are presented in Table 7.

**Table 6:** Morphine concentrations in blood and various tissues. *EC*, enzymatic cleavage; *n.a.*, not available

Case	Femoral blood [mg/l]	Femoral blood (EC) [mg/l]	Heart blood [mg/l]	Liver [mg/kg]	Kidney [mg/kg]	Cerebellum [mg/kg]	Brainstem [mg/kg]	Cerebro-spinal fluid [mg/l]
1	7.96	7.99	14.0	8.66	6.74	1.74	n.a	1.00
2	4.30	4.42	18.8	6.48	6.85	3.67	0.85	3.38
3	5.82	5.91	5.37	3.40	3.57	2.35	1.85	1.32

**Table 7:** Ratio of morphine concentrations in tissue material to femoral blood. *EC*, enzymatic cleavage; *n.a.*, not available

Case	Femoral blood (EC)	Heart blood	Liver	Kidney	Cerebellum	Brainstem	Cerebro-spinal fluid
1	1.00	1.76	1.09	0.85	0.22	n.a	0.13
2	1.02	4.37	1.51	1.59	0.85	0.20	0.79
3	1.01	0.92	0.58	0.61	0.40	0.32	0.23
Literature data [3]	n.a	1.26	2.11	4.39	0.51(brain)		n.a

### Case 1

Lidocaine, cafedrine, rocuronium, fentanyl, midazolam, sufentanil, and duloxetine were detected with general unknown analysis. Quantitative analysis with LC–MS/MS revealed 0.42 mg/l midazolam, 0.0078 mg/l fentanyl, and 0.029 mg/l duloxetine. Except for duloxetine, all substances were administered at the clinic.

**Fig. 1:**
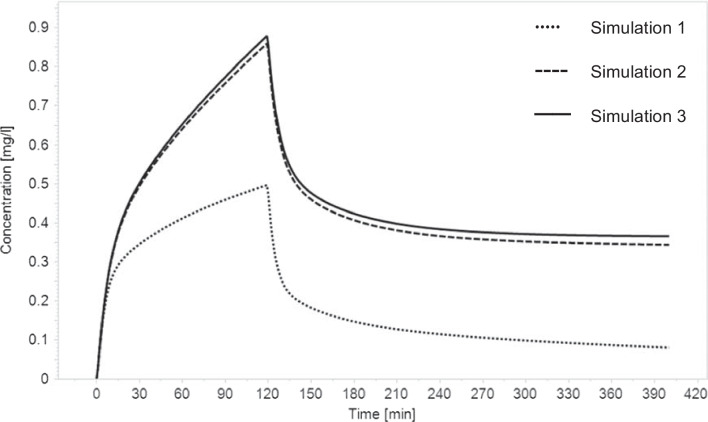
Pharmacokinetic simulation of morphine, exemplified for case 1. Simulation 1: elimination as reported. Simulation 2: no hepatic metabolism. Simulation 3: neither hepatic metabolism nor renal elimination

The PBPK model parameters for morphine given in Table 4 together with the individual parameters given in Table 5 and the administered morphine dosage (Table 1) were applied to predict morphine concentrations to be expected at the time of death. The predicted free morphine concentration was 0.47 mg/l. Disabling metabolism of morphine, the predicted concentration rose to 0.79 mg/l. Disabling both, hepatic metabolism and additional renal clearance, the predicted blood concentration was 0.81 mg/l. These simulations are shown in Fig. 1. In comparison, the actual blood levels were 9.8 times higher than the highest value obtained in these simulations.

### Case 2

General unknown analysis of femoral blood revealed nicotine, cotinine, lidocaine, rocuronium, fentanyl, and diazepam. Quantitative LC–MS/MS analysis of femoral blood serum revealed 0.073 mg/l diazepam and 0.0026 mg/l fentanyl. All substances were documented at the clinic.

Using the morphine dosage (Table 2) and the parameters given in Tables 4 and 5, simulated morphine concentration was 0.73 mg/l. Disabling hepatic metabolism, the predicted concentration was 2.19 mg/l, and without metabolism and renal elimination, it was 2.30 mg/l. However, the analysed morphine concentration was still 1.9 times higher.

### Case 3

General unknown analysis of femoral blood revealed ketamine, morphine, melperone, cafedrine, lidocaine, midazolam, quetiapine, lorazepam, cefazoline, and fentanyl. Femoral blood serum was quantified via LC–MS/MS analysis and 0.011 mg/l lorazepam, 0.035 mg/l midazolam, 0.0092 mg/l fentanyl, 0.12 mg/l quetiapine, and 0.62 mg/l melperone were found. Except for quetiapine and melperone, all substances were administered at the clinic.

Simulation of morphine blood concentration with the parameters given in Tables 3, 4, and 5, predicted a morphine concentration of 0.53 mg/l. Without consideration of hepatic metabolism, the predicted morphine concentration was 1.72 mg/l, and without metabolism and renal elimination, it was 1.83 mg/l at the time of death. The analysed concentration was again still 2.5 times higher.

## Discussion

Severe burn injuries are associated with significant morbidity and mortality. Patients with burn injuries as severe as those presented here usually experience a very short survival time and die before they can receive sufficient medical care in a hospital. For this reason, cases as those presented here are uncommon in forensic practice. In all three cases, unusually high morphine concentrations in postmortem femoral blood were detected (Table 6). For the intensive medical treatment of severe pain, potent opioids such as morphine are suitable. The dosage and administration method have to be adjusted based on individual requirements, especially in palliative care. Therefore, there is generally no clinically significant maximum dose limiting adequate analgesia for strong opioids [10–13].

Therapeutic morphine blood plasma concentrations in living patients are within 0.01–0.1 mg/l [14]. Ketola et al. [15] published a summary statistic for drug concentrations in postmortem femoral blood representing all causes of death. For morphine (*n* = 2134), the upper 97.5th percentile in femoral blood is given as 0.95 mg/l. According to the authors, the upper percentile concentrations indicate possible overdosage levels. The measured concentrations in our cases were consistently higher. Therefore, we aimed to use a pharmacokinetic model to better understand the kinetics of morphine in severely burn injured individuals. Morphine dosage was accurately documented in the clinic, which allows for adequate interpretation of analysed results and morphine administration was necessary for sufficient (palliative) care.

The blood concentrations of morphine predicted by PBPK did not agree with the measured morphine levels. Even if it is assumed that the administered morphine was not metabolized at all and no renal excretion took place, the calculation predicts significantly lower values than were actually measured.

To find explanations for the high morphine concentrations and the poor prediction of the PBPK model, the influence of burn injury and pathophysiologic changes on pharmacokinetics has to be discussed. Burn injuries develop in two phases. Exclusively, the first phase is relevant in our cases, as the individuals died within hours only due to the severity of the burns. In the first phase, which occurs during the first 48 h after thermal injury, burn injury usually results in a distributive shock [16]. Local inflammatory processes are initiated via activation of the Hageman factor (factor XII), the coagulation/fibrinolysis system, the kallikrein-bradykinin cascade, and the complement and arachidonic acid cascades [17]. This results in the release of systemic inflammatory mediators such as histamine, serotonin, neuropeptides, complement proteins, and free radicals [18, 19]. As a consequence, capillary permeability is increased [20, 21], resulting in a transient shift of fluid and albumin into the interstitial space. Consequently, interstitial volume is up to twice as large in the first hours after injury [22], accompanied by hypovolaemia [23, 24], reduced glomerular filtration rate, and urine excretion [25].

The oxidative stress caused by burn injury also depresses cardiac function within a few hours of injury. The decrease in cardiac function and relative hypovolaemia along with low blood flow caused by vasoconstriction affects perfusion of tissues and organs, including the heart, lungs, liver, and gastrointestinal tract—augmenting tissue and organ dysfunction and damage. The state of shock continues even if hypovolaemia is corrected [26].

Morphine is largely eliminated by hepatic metabolism, and its major metabolites, morphine-6-glucuronide (M6G) and morphine-3-glucuronide (M3G), are mainly eliminated by renal excretion. M3G is not an active substance, whereas M6G is suspected to contribute significantly to the analgesic effect of morphine [27, 28]. It is reasonable to assume that decreased hepatic blood flow results in a lower glucuronidation rate of morphine. In all three cases, the morphine concentrations were not increased at all or at most very slightly after glucuronide cleavage as shown in Table 7. This finding indicates that at most only a small fraction of morphine was metabolized to M3G and M6G, which results in elevated free morphine blood concentrations.

Another explanation for the low increase after glucuronide cleavage could be that the glucuronides already broke down or were cleaved by microorganisms in the time interval between death and internal examination. In blood samples from living individuals stored at 4 °C for 181 days, M3G and M6G were shown to be stable [29]. In postmortem blood, bacteria can be a source of glucuronidase activities [29], which might result in low concentrations of morphine glucuronides. Usually, enzyme activities show a rise in metabolism at higher temperatures. It is therefore expected that the degradation of morphine glucuronides to morphine by enzymatic cleavage is also temperature dependent. Morphine glucuronides in postmortem blood samples were found to be stable in vitro at − 20 °C [29, 30]. Since the deceased were stored cooled immediately and consequently after death, no relevant degradation of glucuronides that could have increased the concentration of free morphine has to be assumed.

In addition to impairment of hepatic metabolism, impairment of renal excretion of morphine is also likely to contribute to an increase in blood morphine concentrations. Clinical documentation confirms reduced renal clearance in all three cases, indicating a moderately to severely decreased renal excretion [31]. Consistent with the documentation, in all three cases, the urinary bladder was found empty at autopsy, although a total of 1.5 L and 4.2 L isotonic electrolyte solution were administered in cases 1 and 3. Fluid therapy is necessary to counteract the decreased cardiac output, decreased renal, hepatic, intestinal blood flow, and increasing haematocrit [32, 33]. All deceased were catheterized in the hospital, which may provide an alternative explanation for the absence of urine. However, case 1 exhibited only 25 ml urine excretion in the hospital, while case 2 and 3 showed none.

Even though the PBPK model can account for impaired renal elimination and impaired hepatic metabolism, the predicted values are still quite low in comparison to measured morphine values. The simulation underestimates observed morphine concentrations irrespective of metabolism or renal elimination. However, these assumptions can be taken into account, as there was no renal output and little morphine metabolism. Disabling both factors led to a closer fit to observed values in all three cases.

Literature concerning pharmacokinetics of morphine in major burn injuries is scarce. Perry et al. [34] found no significant alterations in long-term intravenous infusion in eight patients with 6–37% BSA second-degree and third-degree burn injuries, neither did Perrault et al. [35] in five patients with major or critical burns, defined as first degree covering > 75% of BSA plus systemic symptoms, or second degree on > 25% of BSA, or third degree on > 10% of BSA. In contrast, Furman et al. [36] found the volume of distribution and clearance (Cl) to be 1.5- and twofold lower, respectively, and t_1/2_ to be 1.5-fold longer in seven burn patients (25–85% BSA, mean 46% BSA). However, it should be noted that both works give data at continuous long-term application. Thus, these patients are probably already in the 2nd phase of changes after burn injuries. In this phase, a hypermetabolic state is typically observed (flow phase) [26]. In addition, the burn injuries described from both authors appear less severe than in the presented cases.

Limited distribution in burn shock is further likely to have had a relevant influence on morphine distribution. The low distribution of morphine from the blood to the organs can be followed by the ratio of morphine concentration in tissue material to femoral blood morphine concentrations (Table 7). The ratios are lower compared to data published by Ketola and Kriikku [3] for abdominal organs and roughly comparable to brain tissue.

Case 1 received morphine for 2 h and 37 min and case 3 for 1 h 59 min, whereas the application in case 2 lasted for 5 h and 9 min. The duration of morphine administration and the tissue/blood ratios in cases 1 and 3 are comparable. In case 2, similar results are found, although not as pronounced as in cases 1 and 3. It can be assumed that the longer application time led to a somewhat more intensive distribution than in the other two cases. These results suggest that the high morphine concentrations in femoral blood, in addition to decreased metabolism and excretion, are also a consequence of reduced distribution of the drug from this compartment into tissue.

The low distribution of morphine in burn shock may close the gap from the PBPK predicted morphine concentration and the here measured concentrations.

Case 1 shows the highest concentration of morphine in blood (Table 5). In this case, the infusion was not stopped until 18 min after the moment of death. The particularly high concentrations in this case can therefore partially be attributed to incomplete distribution of the amount of morphine applied after the onset of circulatory stop and death.

Some doubts may be raised regarding the adequacy of this approach. We used a PBPK model to simulate morphine, but there is limited knowledge about pharmacokinetic modelling of morphine in burn injury [37, 38]. The limitations of applying simulations in forensic toxicology arise from the scarcity of specifically tailored physiologically based pharmacokinetic (PBPK) models for forensic purposes. Validation poses a challenge as comparisons with biopsy, and postmortem data are often constrained. Postmortem changes, including altered drug-tissue interactions, introduce complexities, leading to variability in measured concentrations. Uncertainties related to the time elapsed between death and blood concentration measurement further impact data interpretation. Polypharmacy’s role in drug overdose complicates assessments, and there is a notable absence of PBPK models addressing drug-drug interactions in forensic scenarios. While scarce, existing forensic PBPK models, primarily focused on a potential morphine overdose, highlight the need for additional model development and validation using diverse datasets [39]. Therefore, quantitative comparison of these results should be made with caution. Nevertheless, this approach provides an initial estimation of the influence of burns on morphine pharmacokinetics during the acute trauma phase and may help in interpretation of toxicological findings in burn fatalities from now on.

## Conclusion

In conclusion, unusually high morphine concentrations in femoral blood after therapeutic dosage of morphine together with relatively low concentrations in tissue in three fatal cases after severe burn injury were reported. Pharmacokinetic simulations were able to show that the findings cannot be explained solely by lower morphine metabolism and reduced renal clearance. The morphine tissue/blood ratios allow to assume that burn shock in particular leads to altered pharmacokinetics, namely reduced distribution of morphine. The results of this study contribute to a more substantiated interpretation of morphine concentrations in postmortem specimens, especially in cases with severe burn injury. These findings should be taken into consideration when interpreting morphine concentrations in similar cases. However, the assessment of morphine concentrations in fatalities remains a challenging task.

## Data Availability

The data underlying this article are available in the article.
